# Biocidal effects of a wipe-down procedure using common veterinary cleansers on microbial burden within working canine exterior coats

**DOI:** 10.3389/fvets.2023.1219249

**Published:** 2023-07-24

**Authors:** Erin B. Perry, Dakota R. Discepolo, Stephen Y. Liang, Maurnice Scott, Kyleigh Williamson, Kelly S. Bender

**Affiliations:** ^1^Department of Animal Science Food and Nutrition, Southern Illinois University, Carbondale, IL, United States; ^2^Department of Emergency Medicine and Division of Infectious Diseases, Department of Medicine, Washington University School of Medicine, St. Louis, MO, United States; ^3^School of Biological Sciences, Microbiology Program, Southern Illinois University, Carbondale, IL, United States

**Keywords:** canine, decontamination, wipe-down, biocidal, antimicrobial

## Abstract

**Introduction:**

Recent work demonstrating reduction of aerosolized contamination via a wipe-down procedure using common veterinary antiseptics offers promise regarding health concerns associated with cross-contamination from working canines to humans. While mechanical reduction can be achieved via a wipe-down procedure, the biocidal impact on flora within the exterior coat is unknown.

**Methodology:**

This study assessed the biocidal impact of antiseptics on the exterior bacterial community of the canine. Lint-free towels were saturated with 2% chlorhexidine gluconate scrub, or 7.5% povidone-iodine scrub diluted at a 1:4 ratio. Treatments were rotated across the dorsal aspect of kennel housed Foxhounds (*n* = 30). Sterile swabs were collected in triplicate prior to, and following wipe down, stored in Amies solution at 4°C, plated onto nutrient agar and reduction in colony forming units (CFU) was measured across both treatments. Statistical analysis utilizing PROC GLM examined effects of treatment (*p* ≤ 0.05). Molecular analysis of the 16S rRNA gene was completed for 3 hounds.

**Results:**

Reduction in CFU was measured (*p* < 0.001) for both antiseptics. Qualitative molecular data indicated that both antiseptics had a biocidal effect on the dominant microbial community on the exterior coat with gram-positive, spore-forming taxa predominating post-treatment.

**Conclusion:**

Effective wipe-down strategies using common veterinary cleansers should be further investigated and incorporated to safeguard working canine health and prevent cross-contamination of human personnel.

## 1. Introduction

Cleaning strategies in healthcare facilities often include wipe-down procedures using detergents and disinfectants with biocidal activity to reduce microbial contamination of high-touch surfaces and prevent fomite transmission of pathogens to patients (2). As animal-assisted therapy for patients and healthcare personnel (HCP) has become more commonplace, the exterior coat of working canines represents a unique high-touch fomite surface not well-understood in infection prevention. While expert guidelines emphasize hand hygiene before and after each animal contact and describe how to prepare the animal prior to visiting a healthcare facility (e.g., bathe with a mild, hypoallergenic shampoo if malodorous or visibly soiled), recommendations regarding disinfection of the animal’s coat between interactions with patients and HCP are lacking. Limited data are emerging regarding the efficacy of wipe-down procedures involving working dogs (including therapy and service dogs) (1, 3). However, the biocidal activity of these methods has not been well-characterized.

Outside of healthcare, working canines are frequently exposed to pathogens that can be harmful to the animal, its human handler, and others the animal may encounter. For example, disaster canines frequently deploy to contaminated environments with high levels of fecal coliforms (4, 5), often in the setting of compromised sewage systems (6). Recent emphasis on canine decontamination and hygiene has increased awareness of the risk of cross-contamination to humans (1, 7, 8). While canine-to-human cross-contamination with oil-based agents despite standard decontamination procedures has been described (8), the risk of microbial cross-contamination has not been well-characterized. Recent work identifying the shared microbiota of canines cohabitating with humans suggests such transfer is likely (9). Evidence-based canine decontamination strategies are needed to mitigate microbial contaminants present on the exterior coat of the working canine. Common bathing procedures utilized for canine decontamination are resource-intensive, impractical, and may result in damage to canine skin if repeated frequently (10, 11). A simple, practical wipe-down procedure would be useful in preventing fomite transmission of pathogens from the canine exterior coat to humans and their surrounding environment.

## 2. Materials and methods

### 2.1. Animals and swab collection

Institutional Animal Care and Use approval (# 19–031) was obtained from Southern Illinois University prior to the initiation of this study. Working canines (Foxhounds, *n* = 30) housed in similar outdoor kennel facilities were utilized in this study. Study participants included intact female (*n* = 10), intact male (*n* = 11) and neutered male (*n* = 9) dogs. All participants were considered “ideal” weight (BCS 4–5) and ranged from 2 to 11 years of age. Routine vaccinations as well as internal and external parasite prevention measures were current for all study participants.

Sterile cotton tipped swabs were utilized for sample collection. Swabs were collected following 30 s of contact time utilizing continuous bidirectional rotation while following the direction of coat growth. Swabs were collected in triplicate prior to and following wipe-down with one of two antiseptics evaluated and stored in Amies transport media (1 mM MgCl_2_ x 6 H_2_O, 1.5 mM KH_2_PO_4_, 8 mM Na_2_HPO_4_, 1 mM CaCl_2_, 2.7 mM KCl, 50 mM NaCl, and 1 g sodium thioglycolate per L). Unused swabs were saturated in sterile deionized H_2_O (prior to storage in Amies transport media) to serve as controls.

### 2.2. Wipe-down procedure

**Figure 1:**
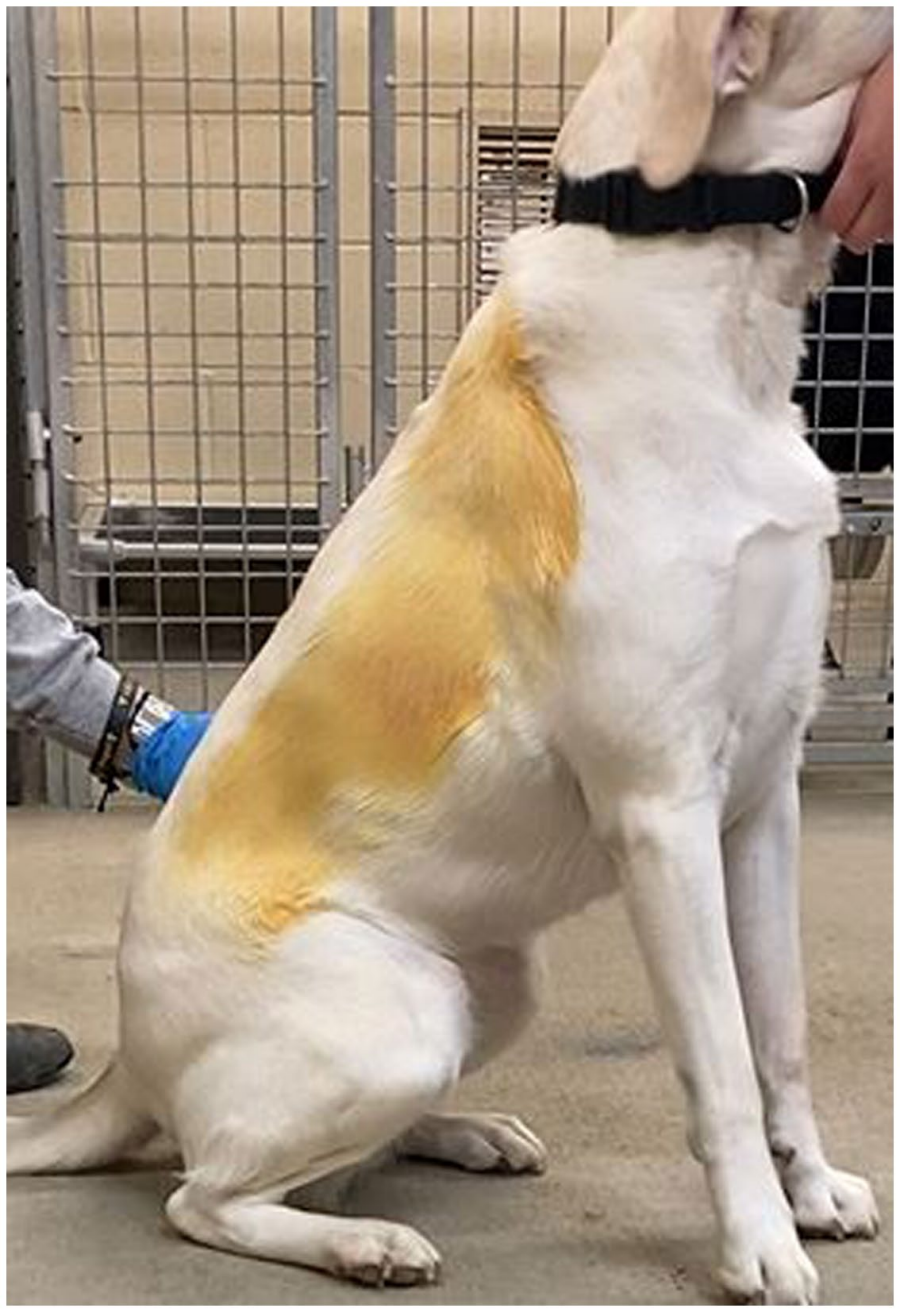
Lateral view of PVD residue remaining after treatment wipe.

Disposable, lint-free towels (Davelen©; Derwood, Maryland) were saturated with 2% chlorhexidine gluconate scrub (CHX) or 7.5% povidone-iodine scrub (PVD) diluted in sterile water at a 1:4 ratio. The dorsal aspect of each canine was divided into left and right segments and treatment wipes rotated between left and right sides for each dog (see Figure 1).

Each canine was wiped down using CHX on one side and PVD on the other, alternating sides (left vs. right) with each successive participant. Wipe-down with the disinfectant-saturated towels was applied from the shoulder to the hip. Following this initial wipe-down, a second was applied in the same fashion using a water saturated towel to remove any disinfectant residue.

### 2.3. Bacterial analysis

Biocidal activity was measured quantitatively by colony count (colony forming unit, CFU). 0.1 mL Amies transport medium from each swab collection and transport device was inoculated onto nutrient agar (BD Difco™) plates using the spread plate technique. For swabs resulting in too many colonies to count (TMTC), serial dilutions were performed until a statistically significant number of colonies (30–300 CFU/mL) was obtained. Final colony counts for each of the triplicate swabs (including calculations for the diluted samples) were averaged.

In order to capture in depth microbial data, individual colonies isolated from swabs obtained prior to and following wipe-down for three dogs were submitted for standard polymerase chain reaction (PCR) targeting the bacterial 16S rRNA gene using the universal primers 8F (5’-AGAGTTTGATCCTGGCTCAG-3′) and 1492R (5’-GGTTACCTTGTTACGACTT-3′) (12) and DreamTaq (ThermoFisher), as described by the supplier. PCR cycling parameters consisted of an initial colony matter lysis step of 94°C for 10 min; followed by 30 cycles of 94°C for 1 min, 50°C for 1 min, and 72°C for 1 min; and ending with a final last extension step of 72°C for 10 min. Following agarose gel electrophoresis analysis, amplicons in the size range of ~1482 bp were extracted using the GeneJET (Thermo Scientific) gel extraction kit. The resulting purified DNA was then sent for commercial DNA sequencing using either 515F (5′- GTGCCAGCMGCCGCGGTAA-3′) or U529R (5′- ACCGCGGCKGCTGGC) primers, targeting regions V3-V4 of the 16S rRNA gene. A total of 144 partial 16S rRNA gene sequences were manually analyzed for purity and trimmed of primer sequences. From this initial analysis, 138 trimmed sequences (ranging between ~400–750 bp) were selected for BLASTn analysis (13). For each analyzed sequence, the BLASTn hit with the highest sequence similarity to a named isolate was recorded.

### 2.4. Statistical analysis

Data entry was performed using Microsoft Excel (Microsoft Corporation, Redmond WA) and data were analyzed using SAS, version 9.4 (SAS Institute Inc., Cary, NC). Significance for all variables of interest was established at *p* < 0.05. The effect of treatment was evaluated using a PROC GLM two-way ANOVA to identify changes in CFU count associated with PRE (untreated) versus POST (treated) counts for each cleanser utilized. Means and ranges of CFU values for PRE and POST values including percent reduction of treatments are reported.

## 3. Results

### 3.1. Bacterial quantification

**Figure 2:**
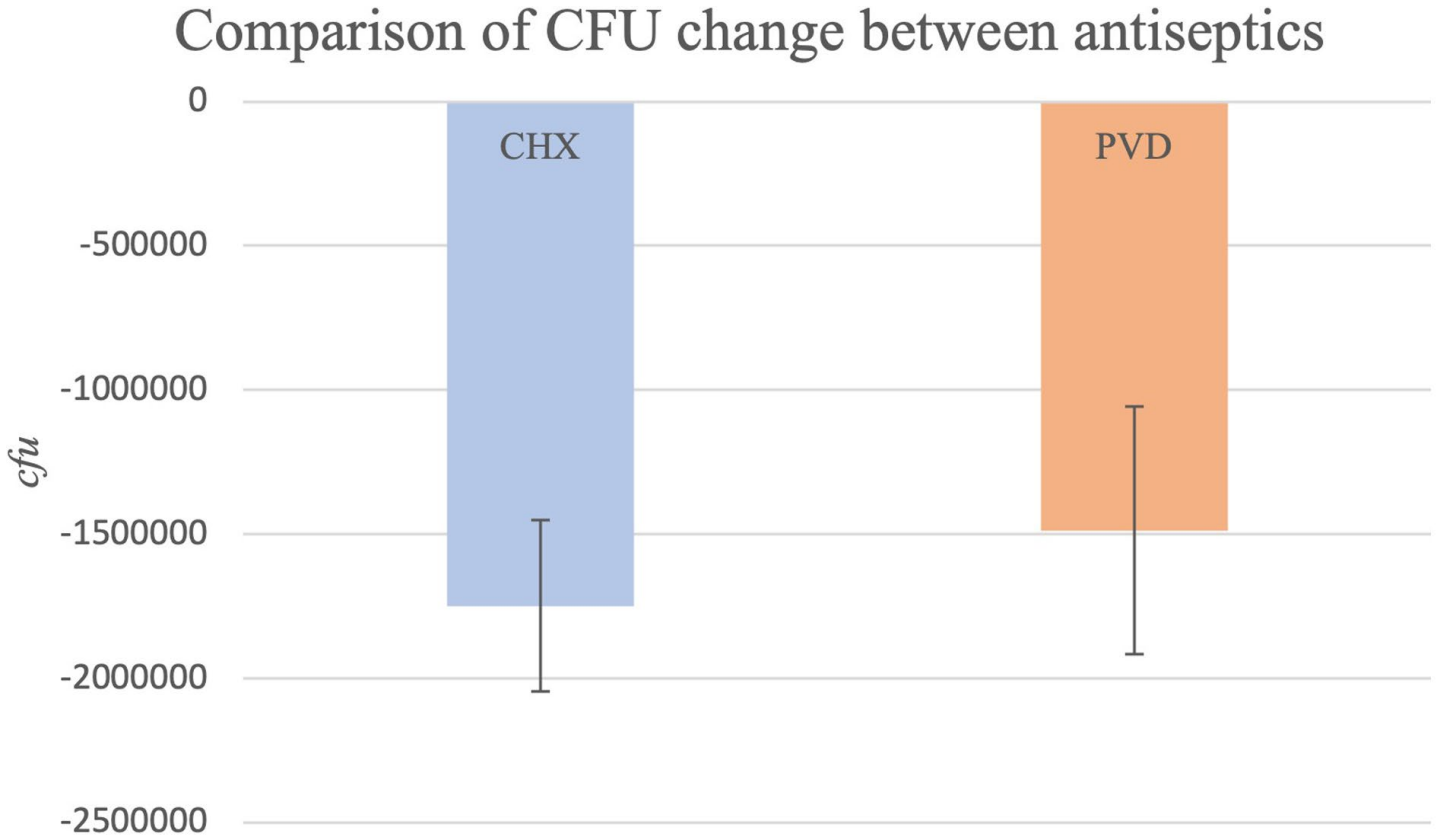
The comparison of mean CFU reduction for both antiseptic treatments (*p* = 0.9192).

Due to CFU counts exceeding the countable range, dilutions were performed to obtain values within the countable range (30–300 CFU/mL) for all PRE samples and 7 POST swabs from the CHX treatment (dog #7- all three swabs; dog #13- all three swabs; dog #30-one swab). No dilution was necessary for POST swabs collected after PVD wipe. CFU values for PRE samples ranged from 1.42 × 10^7^ to 9.03 × 10^3^ (mean: 1.80 × 10^6^), while CFU values for POST CHX and PVD samples ranged from 1 to 5.16 × 10^5^ (average of 3.16 × 10^4^) and 6 to 113 (average of 32), respectively. The overall comparison of PRE values to POST values was highly significant (*p* < 0.0001) indicating efficacy of treatments. The CFU reduction for PVD was −99.98%, while the CFU reduction for CHX was slightly less at −98.61%. However, this difference between treatments was not significantly different (*p* = 0.9192; Figure 2).

### 3.2. Bacterial communities identified

**Figure 3:**
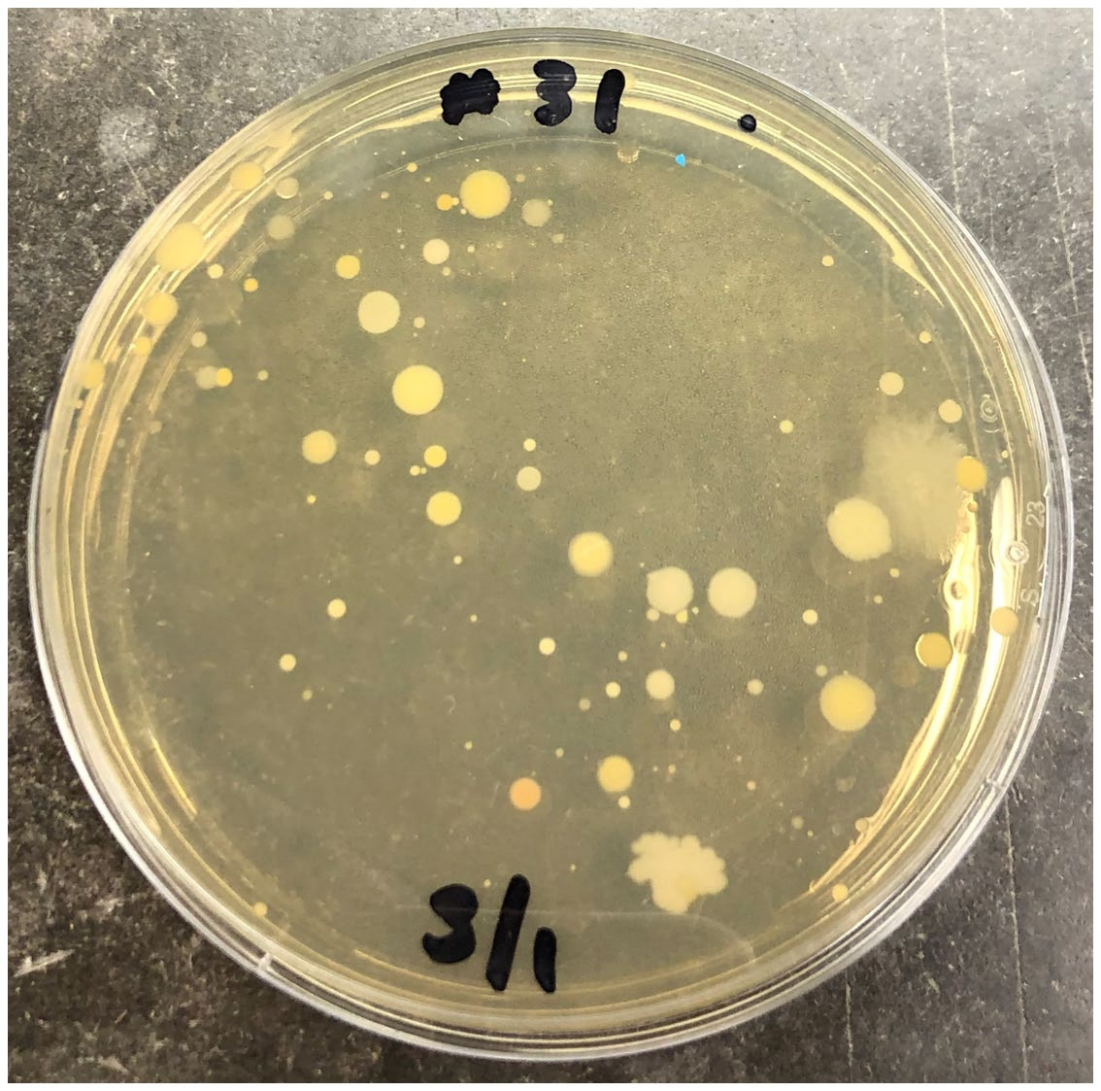
Undiluted sample from swab #31 corresponding to PVD treatment of canine #3 plated on nutrient agar.

An overview of bacterial taxa present on the external canine coat as well as species resistant to the biocidal activity of PVD and CHX wipe-down was determined using a molecular approach targeting the bacterial 16S rRNA gene in colonies obtained from canine participants #17, #19, and #21. Due to the significant biocidal activity observed with both antiseptics studied, few colonies were obtained from these samples (Figure 3).

**Figure 4:**
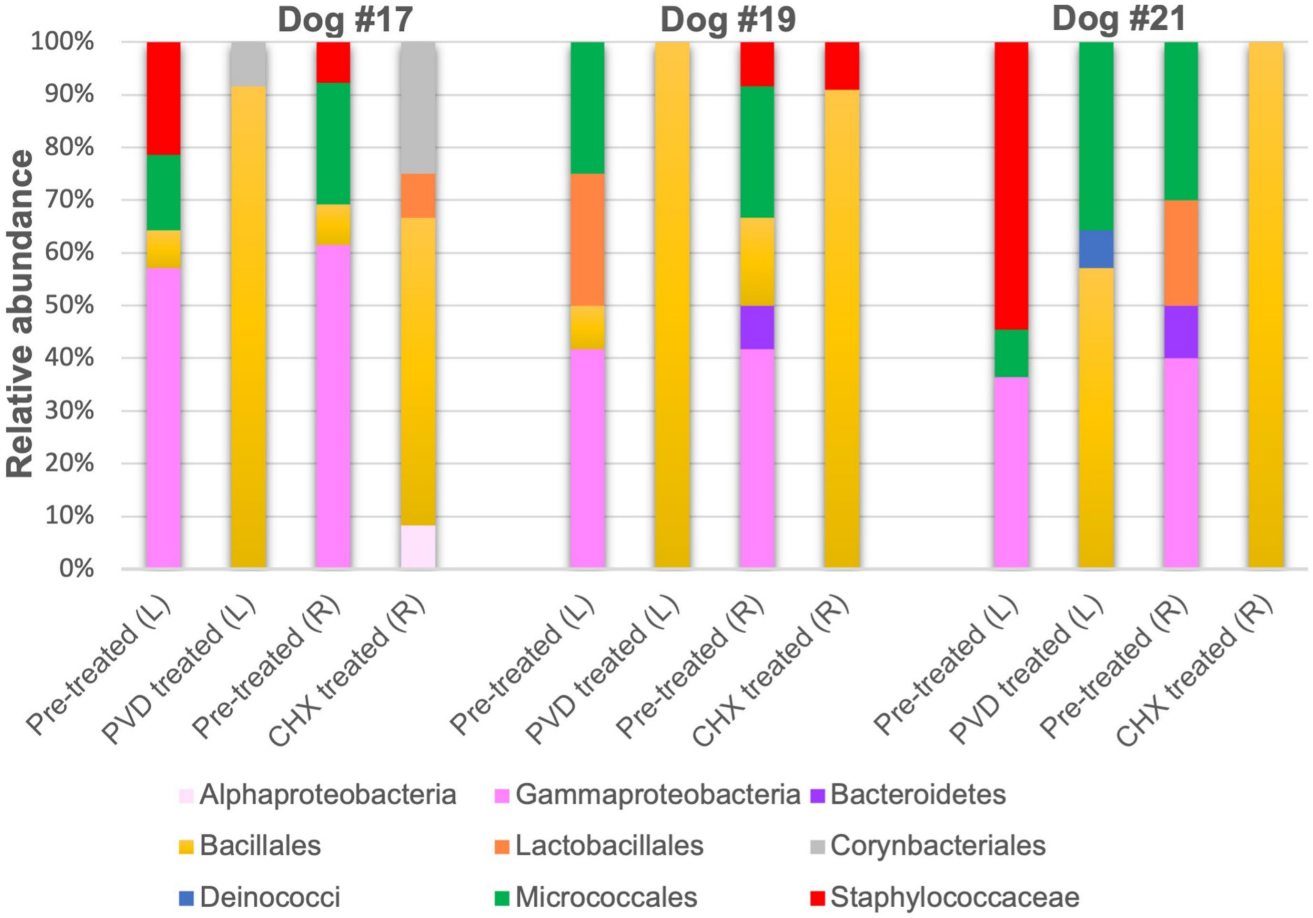
Relative sequence abundance of bacterial orders determined from 16S rRNA gene analysis of colony forming units. PVD: 7.5% povidone-iodine, CHX: 2% chlorhexidine, L: left side, R: right side.

Only 12 colonies per condition for each of the three canines were subjected to PCR targeting the 16S rRNA gene and subsequent sequencing. Because of the strong biocidal activity of CHX on canine #21, extra plating had to be performed to obtain 12 colonies for 16S rRNA gene analysis. The raw sequence analysis from the analysis of 138 sequences (6 sequences were not of good quality and removed) can be found in Supplementary Material. An overview of the orders detected in the swab samples for the three dogs are presented in Figure 4.

Although gram-negative bacteria of the class Gammaproteobacteria were identified in PRE samples from all three canines, both PVD and CHX wipe-down effectively eliminated these taxa. In contrast, POST wipe-down samples for both PVD and CHX were dominated by members of spore-forming Bacillales. This order was detected at a much lower percentage from PRE samples (7.4% for participant #17, 12.5% for participant #19, and undetected for participant #21; Figure 4 and Table 1).

**Table 1:** Taxonomic assignment of most abundant bacterial 16S rRNA gene clone sequences.

Order	Genus/species	GenBank Accession/average % identity match	% of sequences per treatment per animal*^a^*		
			#17	#19	#21
Pre-treated assignments					
Micrococcalles	*Glutamicibacter bergerei* HMF3875	KT983988.1/100%	–	–	14.3
Micrococcalles	*Glutamicibacter species* IR2A07	MK841225.1/99.8%	3.7	13.0	4.8
Lactobacillales	*Carnobacterium inhibens* 96E2	MT032346.1/99.3%	–	13.0	–
Staphylococcaceae	*Staphylococcus equorum* JZ RK-17	MH119700.1/100%	–	–	9.5
Staphylococcaceae	*Staphylococcus equorum* LS220	MT409914.1/100%	11.1	–	14.3
Gammaproteobacteria	*Psychrobacter faecalis* NC7 16S	MT269580.1/99.9%	29.7	26.1	4.8
Gammaproteobacteria	*Psychrobacter maritimus* JM52	MN758812.1/99.9%	11.1	–	–
Gammaproteobacteria	*Psychrobacter submarinus* QS172	MK439598.1/100%	14.8	4.3	–
Post-treated assignments					
Micrococcalles	*Kocuria palustris* RP1	MH141481.1/99.1%	–	–	8.0
Corynbacteriales	*Rhodococcus equi* p67_A11	Q831084.1/99.5%	8.3	–	–
Bacillales	*Bacillus altitudinis*	MT627439.1/100%	–	11.1	–
Bacillales	*Bacillus thuringiensis* GR007	CP076539.1/99.6	–	-	8.0
Bacillales	*Paenibacillus* species 7B-648	KF441697.1/99.2%	–	11.1	–
Bacillales	*Priestia megaterium* FORCN119	MW363319.1/100%	12.5	11.1	–
Bacillales	*Priestia megaterium* S2	CP051128.1/99.8%	8.3	–	–
Bacillales	*Priestia megaterium* UIS0181	MT178181.1/100%	16.7	5.6	–
Bacillales	*Sporosarcina globispora* LMTK33	KY614182.1/99.8%	–	–	16.0
Bacillales	*Sporosarcina* species A9	MN746652.1/99.6%	–	–	12.0

a Relative abundance of specific sequences detected at >5% for at least one animal.

**Figure 5:**
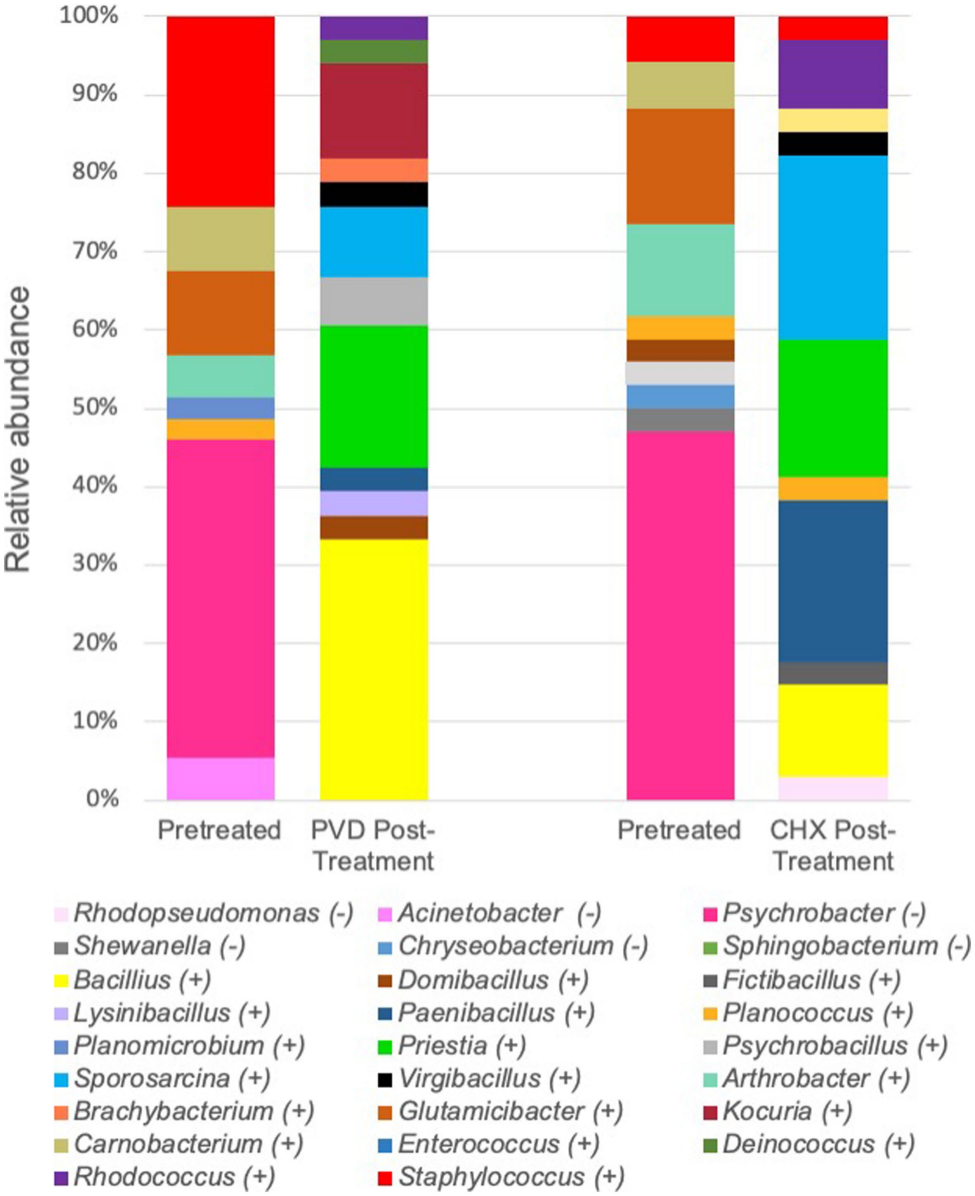
Combined relative sequence abundance of bacterial genera determined from 16S rRNA gene analysis of colony forming units. PVD: 7.5% povidone-iodine, CHX: 2% chlorhexidine, (*−*): gram-negative, (+): gram-positive.

For a more specific analysis of relative abundance at the genus level, the sequence analysis of PRE and POST samples from dogs selected randomly (#17, #19, and #21) was combined (Figure 5). This analysis indicated that PRE samples were dominated by members of the gram-negative genus *Psychrobacter* (~43.7% relative abundance), a psychrotolerant (cold temperature tolerant) bacterium. No traditionally pathogenic genera were identified in PRE samples; *Staphylococcus* sequences detected were most closely related to the *equorum* species (~15.5% relative abundance; Figures 4, 5 and Table 1). Bacterial communities identified in POST PVD and CHX wipe-down samples possessed similar profiles in that gram-positive spore formers predominated (~80.6% relative abundance of *Bacillus*, *Fictibacillus*, *Deinococcus*, *Domibacillus*, *Lysinibacillus*, *Paenibacillus*, *Priestia*, *Psychrobacillus*, *Sporosarcina* and *Virgibacillus* species; Figure 5). *Bacillus* and *Priestia* species detected were non-pathogenic: *thuringiensis, altitudinis,* and *megaterium* (Table 1). Only one colony from the POST CHX wipe-down samples was related to a gram-negative species (*Rhodopseudomonas*).

## 4. Discussion

A simple disinfectant wipe-down procedure using towels saturated with 2% chlorhexidine gluconate scrub or 7.5% povidone-iodine scrub exhibited significant biocidal activity against bacteria present on the exterior coat of working canines, resulting in 99.98 and 98.61% reduction in CFU, respectively (Figure 2). Molecular characterization of a smaller subset of samples demonstrated shifts in bacterial community composition after wipe-down with CHX or PVD. No pathogenic bacteria were identified. These findings suggest that wipe-down procedures using CHX or PVD are effective in reducing microbial burden and exert selection pressure on resident bacterial flora present on the canine exterior coat.

While a limited analysis of the microbial community present on the external coat of three canines was performed, the microbiome selected for further analysis prior to disinfectant wipe-down was dominated by *Psychrobacter* species (Figure 5; Table 1). *Psychrobacter* are psychrophilic or pyschrotolerant (cold-loving or cold-tolerant) gram-negative bacteria associated with a wide range of animals as well as terrestrial and marine environments. *Psychrobacter* species have recently been detected among canine oral (14), conjunctiva (15), and skin (16) microbiomes. Species within this genus rarely cause disease (17) and were likely selected for due to the winter season in which the samples were collected. It should also be noted that the swabs were stored at 4°C post collection.

Another genus of interest detected in samples obtained prior to disinfectant wipe-down was *Staphylococcus* (Figure 5). However, none of these colonies were closely related to pathogenic *Staphylococcus* species (Table 1). Most of the *Staphylococcus* sequences were most closely related to the *equorum* species, which have been isolated from healthy Labrador retrievers (18). The qualitative results from the pre-wipe samples suggest that the canines possessed similar external coat microbiomes, likely a reflection of an environmentally homogenous study cohort.

Samples obtained after disinfectant wipe-down were dominated by the non-pathogenic aerobic/facultative spore-forming gram-positive genera of *Bacillus*, *Paenibacillus*, *Priestia*, and *Sporosarcina* (Figure 5; Table 1). Specifically, *Priestia* (formally *Bacillus*) *megaterium* and *Sporosarcina globispora* were two detected species that are commonly found in the environment (19). While it has been reported that PVD is more sporicidal than CHX (20), these studies were focused on spore formation in *Bacillus subtilis*. No CFU related to the gram-negative *Psychrobacter* species identified in the pre-wipe samples were detected in the either the PVD or CHX treated samples (Figure 5). However, our study design provided only a snapshot of the external coat microbiome and was not able to determine if bacterial species were differentially targeted by PVD and CHX wipe-down. A more exhaustive microbial community analysis is necessary to determine the bactericidal spectrum of each disinfectant on flora present in the canine exterior coat.

The implications of this work to the working canine community are significant. The benefits of working canines across many disciplines is well documented, including in healthcare settings (21, 22). However, the potential for canine-to-human microbial cross-contamination remains an important concern in infection prevention and evidence-based canine decontamination and hygiene procedures are needed. Therapy dogs, service dogs, law enforcement dogs, and disaster dogs are frequently tasked with work resulting in a high degree of contact with the environment which can lead to human cross-contamination with both canine and environmental flora. Prior work has demonstrated that a simple wipe-down procedure utilizing CHX is effective at reducing exterior coat contamination with aerosolized contaminants (1). More recent work investigated the potential efficacy of CHX as a wipe down decontaminant for canine equipment with significant success using viral surrogates (23).

This work clearly demonstrates a beneficial reduction in canine coat microbial burden following a simple wipe-down procedure. Kennel-housed dogs were utilized in this study in order to identify potential benefits in dogs with outdoor exposure. Future studies should incorporate hospital-based working canines in order to assess impacts to microbes typically present in a hospital environment. Additionally, dogs of different breeds with differing coat types should be assessed for potential differences due to coat morphology.

## 5. Study limitations

Disruption due to the COVID 19 pandemic resulted in lack of access to a larger study population due to travel limitations for study technicians. Future work should include breeds commonly utilized in working disciplines including service and therapy dogs. Additionally, future work should include microbial analysis from various anatomical regions to capture a more comprehensive picture of the entire dermal environment and impacts associated with bathing.

## Data availability statement

The datasets presented in this study can be found in online repositories. The names of the repository/repositories and accession number(s) can be found below: https://www.ncbi.nlm.nih.gov/; OR174799 - OR174935.

## Ethics statement

The animal study was reviewed and approved by the Southern Illinois University Institutional Animal Use and Care Committee. Written informed consent was obtained from the owners for the participation of their animals in this study.

## Author contributions

EP supervised study design, study execution, data collection, data analysis, manuscript writing, and review. DD, KW, and KB contributed to study design, study execution, data collection, data analysis, manuscript writing, and review. SL contributed to study design, data analysis, manuscript writing, and review. All authors contributed to the article and approved the submitted version.

## Funding

EP’s work is supported in part by grant # 0297-A from the American Kennel Club—Canine Health Foundation. Additionally, SL received support through the Foundation for Barnes-Jewish Hospital and the Washington University Institute of Clinical and Translational Sciences which is, in part, supported by the NIH/National Center for Advancing Translational Sciences (NCATS), Clinical and Translational Science Award (CTSA) program (UL1TR002345). DD’s work is supported by the Diversifying Higher Education Faculty in Illinois Fellowship. Other support for this project was provided by the Southern Illinois University, College of Agricultural, Life and Physical Sciences.

## Conflict of interest

The authors declare that the research was conducted in the absence of any commercial or financial relationships that could be construed as a potential conflict of interest.

## Publisher’s note

All claims expressed in this article are solely those of the authors and do not necessarily represent those of their affiliated organizations, or those of the publisher, the editors and the reviewers. Any product that may be evaluated in this article, or claim that may be made by its manufacturer, is not guaranteed or endorsed by the publisher.
